# Sustainability concerns on consumers’ attitude towards short food supply chains: an empirical investigation

**DOI:** 10.1007/s12063-021-00188-x

**Published:** 2021-03-17

**Authors:** Meng Wang, Vikas Kumar, Ximing Ruan, Mohammed Saad, Jose Arturo Garza-Reyes, Anil Kumar

**Affiliations:** 1 Bristol Business School, University of the West of England, Bristol, UK; 2 Centre for Supply Chain Improvement, University of Derby, Derby, UK; 3 London Metropolitan University, London, UK

**Keywords:** Short Food Supply Chains, Five Pillars, Sustainability, Empirical Study, China, Moral Economy

## Abstract

While industrialized agro-food supply systems have gained tremendous success in recent decades, it has been increasingly criticized for its adverse environmental and social impact. Amongst this criticism, Short Food Supply Chains (SFSCs) have emerged as a promising sustainable alternative to the industrialized agri-food supply systems. In recent years there have been some attempts to explore the relationship between SFSCs and sustainability, but these are mostly theoretical discussions and lacks empirical validation. This study, therefore, attempts to provide empirical validation of the SFSCs and sustainability linkages. Additionally, from the theoretical perspective, our work extends the traditional triple bottom line constructs and explores two extra dimensions of sustainability in the food supply chain system, namely, governance and culture, thus exploring five dimensions of sustainability. Furthermore, while SFSCs have proven to improve farmers’ livelihoods and reconnect producers with consumers, little or no attention has been given to understand the consumers' attitudes towards the SFSC practices. Therefore, this study aims to explore the customers’ attitudes towards participating in SFSCs through the concept of a moral economy and personal relationship. Based on the 532 valid responses from Chinese consumers, our study shows that all five pillars of sustainability, moral economy and Chinese relationship have a positive influence on consumers’ participation in SFSCs. With its intuitive benefits, the economic pillar emerged as the most approved factor by the participants. Interestingly our findings show that the social aspect is less prominent than others, which is contrary to existing studies conducted in developed countries.

## Introduction

Owing to the industrial revolution, the agriculture system has been geared towards a supply chain model that can maximize efficiency to reduce consumer costs. This led to the emerging trends of supermarket based globalized agro-food system in the last 20 – 40 years (Gereffi 1994). As indicated by Welch and Graham (1999), the most prominent benefits of this conventional food supply system are the lower food costs and larger food variety. Both are achieved through large-scale production and advanced logistics system. The costs to maintain large scale farming are usually lower, especially when compared with organic farming. This is mainly caused by using synthetic chemical fertilizers. Moreover, the modern logistics system can help to transport farm products to other regions or even countries (Wang 2019) Agricultural products have been the main export goods for many countries, such as China, the US, and Brazil (Silva et al. 2017). It should however be noted that this modern form of food supply system has achieved tremendous success in mitigating the food crisis in recent decades.

However, while these conventional agri-food supply chains have been proved effective and successful, they are increasingly accused of their adverse impacts on health (Llazo 2014) and the environment (Mastronardi et al. 2015). The massive production feature of this traditional food supply system has raised widespread concerns about its unsustainability and harms to the environment, such as excessive land use, pollution of soil and water, and exhaust emissions (Bazzani and Canavari 2013). Additionally, it can also be noted that the conventional food system is more favourable for large farming industries and is hence not suitable for small farmers. Produces from small farms are more difficult to be admitted into the system, which can deteriorate the livelihoods of farmers, especially in developing countries, where industrialised farming is rather rare among ordinary rural farmers. Moreover, with the rapid development of modern society, consumers’ selection of food is not merely limited to maintaining basic living demands, but also considers the safety, environment, and other affiliated values. Therefore, their preferences on conventional supplied food keep deteriorating, as the industrialized food suffers from safety crisis, and tends to have minimal affiliated values. This changing attitude is a growing threat to the maintenance and future of conventional agri-food supply chains. Meanwhile, from the farmers’ perspective, while the lower sale price caused by massive production has been a competitive advantage and granted them substantial revenues in past years, the cost-price squeeze of commodity production and the increasing maintenance costs of massive production have been compressing their economic margin (Berti and Mulligan 2016). The profits of large-scale industrialised farming have been decreasing in recent years, resulting in increased pressure on farmers’ incomes (Renting et al. 2003). Therefore, it can be noted that the preferences of both farmers and consumers on conventional food supply chains are changing in a deteriorating trend.

With the increasing concerns on agriculture sustainability and food safety, several different forms of food supply chain have emerged over the years, which can be categorized as alternative and local food systems (Renting et al. 2003). These newly proposed food supply systems abandon the main features of the conventional food chain, such as massive production and standardized organization (Higgins et al. 2008). The emergence of these new food supply systems originates from the demand for a spatial, economic and social re-localization of the food systems (Goodman and Goodman 2008). A growing trend in developing these new food systems have been witnessed in recent years, which is represented by the growth in selling organic, fair trade, local and quality food through typical instances, such as farmers’ markets, CSAs, farm shops, etc. (Wang et al. 2018; Maye and Kirwan 2010). Owing to their potential in mitigating the sustainability and safety issues and improving livelihoods of farmers, the interest in these new forms of food supply chains have surged among academic, campaigning and policy-making circles in the last two decades (Owen 2014).

Among these newly emerged forms of food supply systems, Short Food Supply Chains (SFSC) is identified as a prominent sustainable practice (Kumar et al. 2019; Wang et al. 2018; Marsden et al. 2000). It operates like a local food system and short-circuits the traditional long food supply chains. The ‘short’ not only refers to the close proximity in geography, but also the social relations between producers and consumers (Renting et al. 2003; Aubry and Kebir 2013; Falguieres et al. 2015). It was originally proposed as examples of farmers to show resistance to the modernization of the food system (Van der Ploeg et al. 2000), and received resurgence interest in recent decades (Ilberz and Maye 2005; Kneafsey et al. 2013; Sellitto et al. 2018; Kumar et al. 2019). While modern research on SFSCs can be traced back to 2000 (Marsden et al. 2000), there still has been no consensus on a unified definition of this concept, with more than 11 different definitions proposed in existing studies (Wang et al. 2018). Nonetheless, it can be noted that SFSCs generally refers to any forms of re-joining farmers with consumers, with a minimized number of intermediaries (Ilberz and Maye 2005). Instead of solely exchanging a product, this direct connection between producers and consumers shares additional information about knowledge, value, the meaning of the product, and producer and consumer themselves (Marsden et al. 2000).

Although SFSCs have received rapid increasing research interest in the last two decades, it should be noted that the majority of existing studies on this newly emerged form of food supply systems were conducted in developed countries (Kumar et al. 2019). Owing to the dominant importance of agriculture in developing countries, it would hence be beneficial to investigate the adoption of SFSCs in a developing country context. Moreover, while SFSCs have proven to improve farmers’ livelihoods and reconnect producers with consumers (Deller et al. 2017; Singh 2013), little or no attention has been given to understand the consumers' attitudes towards the SFSC practices. Therefore, this study aims to explore the customers’ attitudes towards participating in SFSCs in a developing country context.

This paper is structured into six sections. Apart from the introduction, a focused literature review on existing studies of SFSCs and sustainability relationship is presented in Section 2. The theoretical framework and adopted methodology are presented in Section 3 and 4, respectively. Afterwards, Section 5 presents the findings and discussions. Finally, Section 6 concludes this study by highlighting the limitations of the proposed study and suggesting future research directions.

## Short Food Supply Chains (SFSCs) and sustainability

As an alternative to conventional food supply systems, a paramount focus of existing SFSCs research is to investigate its linkage with sustainability. With the widely acknowledged three pillars of sustainability, many researchers have conducted a study on sustainable supply chain management focusing on either a specific one or a mix of these pillars (Khan et al. 2021; Sharma et al. 2020; Kumar et al. 2019). Among these three pillars, social sustainability was the most extensively evaluated dimension. For instance, Hinson and Bruchhaus (2008) explored consumer preferences for locally produced strawberries through a survey of 309 consumers. They found that consumers show more trust in local products and are willing to support local growers. Moreover, the improved quality and enjoyable social atmosphere of these short chains were also confirmed. Giampietri et al. (2016, 2018) conducted two continuous studies to investigate the motivations of consumers’ purchasing behaviour in SFSCs. A questionnaire survey containing 112 university students was conducted in the first study. In the following study, the survey was extended to 260 participants with different backgrounds. They found that the direct interactions in SFSCs can reinforce consumers’ trust in food security and quality and increase consumers’ involvement in local development. A similar finding was also obtained by O’Kane and Wijaya (2015). They investigated farmers’ motivations to join Farmers Market (FM) and its linkage to social sustainability. Semi-structured interviews, field observations and document analyses were conducted to gather data from relevant parties of farmers’ market. They found that farmers felt more empowered and equitable with farmers’ market, and consumers also showed more trust with the high-quality food products. The significance of the social interaction of SFSCs is also confirmed in the literature review conducted by Ashtab et al. (2020). Apart from the social benefits introduced by direct interactions, gender equality was also investigated in SFSCs. Zirham and Palomba (2015) focused on female agriculture entrepreneurship in SFSCs. Four case studies were implemented through open and semi-structured interviews. They found that female agriculture entrepreneurship in SFSCs can benefit from improved food security and a more pleasant shopping atmosphere. Meanwhile, two case studies were conducted using open interviews in the following study, which aimed to investigate women’s role in SFSCs (Zirham and Palomba 2016). It was found that female features can effectively promote direct sale businesses, and hence confirmed the importance of gender equality in SFSCs.

Unlike the widely acknowledged improvements in social sustainability, research on the linkage between SFSCs and economic sustainability is relatively limited. For Farmer Markets type of SFSCs, Watts et al. (2011) investigated the geography of local food activity through a database analysis of 723 enterprises. Their study found that food re-localization can help to retain added values in local areas and hence facilitate economic development. Another study was conducted by Benedek et al. (2017), which compared conventional markets and farmer markets in Hungary. Based on a survey among 156 markets, they reported that farmers within farmer markets are more open to cooperation and tend to be higher educated. Both studies found that direct interactions can help to regain the profit shared by intermediates in conventional food supply systems and facilitate economic development of local areas. The pleasant social atmosphere can be retained as added values to the food products. While the economic sustainability of FMs and direct sales is obvious, there is some controversy over Community Supported Agriculture (CSA). Balázs et al. (2016) examined the CSA movement in Hungary through semi-structured interviews, consumer survey and secondary data analysis. Meanwhile, Janssen (2010) explored the operation of CSA through interviews with eight CSA growers. While Balázs et al. (2016) confirmed that CSA can improve farmers’ financial situation and facilitate local economic development, both of them and Janssen (2010) found the scaling up of CSA can be a major challenge. This is because the investment of CSA is much greater for hiring external labours. Thus, it can be a tough decision for growers to adapt to this form of SFSCs. Moreover, the empirical evidence of the return on investment for CSA is quite limited. The ambiguous effect of economic sustainability was also confirmed by Charatsari et al. (2020). During a comparative study, they invited 33 farmers to participate in SFSCs and 38 farmers involved in the conventional food system in Greece. While they agreed that farmers treat these SFSC schemes as an opportunity to increase profit (Zhang et al. 2019), it was found the potential economic benefits of participating in SFSCs is not the main motivation of their participation.

Similar to the economic pillar, existing studies investigating the environmental dimension is also rather limited. The first located study was conducted by Hara et al. (2013), which investigated the local food movement in Japan, with a specific focus on vegetables in the Osaka city region. A multi-scale and a scenario analysis were implemented to examine the energy consumption. Meanwhile, interviews were also conducted at three farmers’ markets. It was found that while these farms tended to be of low profit, they can effectively reduce energy consumption and hence improve environmental sustainability. Another study was implemented by McClenachan et al. (2014). They compared the environmental impacts of community-supported fisheries (CSFs) and industrial fisheries. Data were collected from 15 CSFs in North America. They found CSFs have much smaller environmental impacts than industrial fisheries, especially for the carbon footprint. While CSFs were confirmed to be a more environmentally sustainable alternative to industrial fisheries, they also indicated the scaling up of CSFs will be a major challenge. Tasca et al. (2017) also evaluated the environmental impact of SFSCs. They used life cycle assessment (LCA) to examine the environmental impacts of organic and integrated farming and their distribution chains. Farm owners, company managers, farmers and consumers were interviewed to collect relevant data. They found that the abandon of disposable packing and industrial processing indirect distribution can effectively reduce environmental impacts by 20% to 48%. Nevertheless, they also indicated that additional improvements, such as better fertilization practices, are still needed to further improve the environmental sustainability of SFSCs. A more recent study by Kiss et al. (2019) using a content analysis of 128 publications reports that while SFSCs can normally reduce environmental impacts caused during transportation and production, these effects cannot be generalised across all kinds of SFSCs, and the performance can be affected by many external factors, such as the spatial location, type, and individual attitudes of involved participants.

From the reviewed literature, it can be noted that SFSCs can benefit the social, economic and environmental dimensions of sustainability. The social benefits of SFSCs are most widely acknowledged. Meanwhile, although economic and environmental benefits are relatively limited, it was still found that SFSCs can mitigate the price squeeze and increase farmers’ incomes by regaining the profits shared by intermediates in conventional food supply systems. Moreover, SFSCs can also improve biodiversity, adopt more eco-friendly production methods, and reduce environmental pollutions. However, it should be noted that most existing studies were conducted in developed countries. As SFSCs can improve food security and increase farmers’ profits, it would hence be beneficial to investigate SFSCs in the developing countries context.

## Theoretical conceptualization and hypothesis development

### Two additional dimensions of sustainability and SFSCs

In the previous section, it was evident that SFSCs is closely related to the three pillars of sustainability (economic, environmental and social dimensions). However, the five dimensions of sustainability were proposed as an extended form of traditional three-dimensional architecture, partially due to the increasing public concerns on the future of humanity (Bervar and Bertoncelj 2016). Hence in addition to the widely acknowledged three dimensions of social, economic and environmental, cultural and governance dimensions have been added that are receiving increasing recognition for their unique linkage and contribution to sustainability.

First proposed in 1995, cultural sustainability was originally categorized under the social pillar but has been increasingly considered as an additional sustainability pillar (Soini and Birkland 2014). Culture itself can be defined as a set of beliefs, morals, methods, and a collection of human knowledge that is dependent on the transmission of these characteristics to younger generations (Merriam-webster 2017). In the case of the SFSCs study, the cultural dimension mainly focuses on human knowledge and beliefs towards the local food and its networks. Some studies have confirmed the cultural benefits of SFSCs. For instance, Sage (2003) found the direct interactions in SFSCs can improve consumers’ belief in the local community. A similar feature was also confirmed by Giampietri et al. (2016, 2018).

Along with cultural sustainability, governance has also attracted increasing attention in recent years, and some researchers have proposed it as an independent sustainability pillar (Biermann et al. 2014; Jitmaneeroj 2016). Although it hasn’t been officially admitted as the fifth sustainability pillar, its importance in delivering the Sustainable Development Goals has been declared by many researchers (Biermann et al. 2014; Williams et al. 2018). FAO (2017) confirmed the necessity of creating a governance system to measure, monitor, and guide sustainable agriculture. Governance mainly concerns the authoritative steering of social processes. Both governmental and non-governmental actors, such as civil societies, partners, and other private entities, are usually involved in governance activities, which can occur at both local and international levels. According to Khan et al. (2021), regulatory policies and aligned government’s sustainable policies are vital factors that motivate the implementation of sustainable practices in supply chain operations.

### Five dimensions of sustainability and hypotheses Development

In the analysis of social SFSCs practices, Giampietri et al. (2016, 2018) found that the direct interactions in SFSCs can reinforce consumers’ trust in food security and quality and increase consumers’ involvement in local development. A similar finding was also obtained by O’Kane and Wijaya (2015). Moreover, O’Kane and Wijaya (2015) found that farmers could feel more empowered and equitable in Farmers Markets (FMs), a typical face-to-face category of SFSCs. Apart from the social benefits introduced by direct interactions, gender equality was also investigated in SFSCs. Two continuous studies by Zirham and Palomba (2015, 2016) explored the females’ role in SFSCs, they found that female features, such as high responsibility and good social manners, can improve food security and provide a more pleasant shopping atmosphere. Moreover, as a form of the local food system, SFSCs can also provide food with improved security to more low-income people (Nonini 2013). Meanwhile, a positive correlation was also found between the density of FMs and Italian adults’ Body Mass Index (BMI), indicating that FMs can provide higher quality food products (Bimbo et al. 2015), and hence promote a healthy diet (Jarzębowski et al. 2020). Based on these discussions, this study proposes the following hypothesis:

H1a. SFSCs positively influence social sustainability performance.

Unlike the widely acknowledged improvements in social sustainability, research on the linkage between SFSCs and economic sustainability is rather limited. Studies focusing on FMs found that the direct interactions can help to regain the profit shared by intermediates in conventional food supply systems and facilitate economic development of local areas (Watts et al. 2011; Benedek et al. 2017). A study by Benedek et al. (2017) also found that farmers within FMs are more open to cooperation and tend to be higher educated. Thus, they can benefit more through direct interactions with customers, and the pleasant social atmosphere can be retained as added values to the food products. While the economic sustainability of FMs is obvious, there is some controversy over Community Supported Agriculture (CSA). While Balázs et al. (2016) confirmed that CSA can improve farmers’ financial situation and facilitate local economic development, both Balázs et al. (2016) and Janssen (2010) found the scaling up of CSA can be a major challenge. This is because the investment of CSA is much greater for hiring external labours. Thus, it can be a tough decision for growers to adapt to this form of SFSCs. It should be noted that the potential increased costs for small scale production are not fully evaluated. However, due to limited empirical studies and conflicting results, the study proposes the following hypothesis:

H1b. SFSCs positively influence economic sustainability performance.

As SFSCs emphasises local production and consumption, there is less movement of the food products compared to industrialised agricultural production. Hence it is likely that in SFSCs will have less environmental impact. Hara et al. (2013) examined the energy consumption of vegetables in the Osaka city region, where they found that the local food movement can effectively reduce energy consumption. Meanwhile, McClenachan et al. (2014) compared the environmental impacts of Community Supported Fisheries (CSFs) and industrial fisheries. CSFs were confirmed to be a more environmentally sustainable alternative with a much smaller carbon footprint. Similar findings were also obtained from the workshops conducted by Jarzębowski et al. (2020). They found that participating in SFSCs can help to reduce food waste and food miles. Moreover, Tasca et al. (2017) found that the abandonment of disposable packing and industrial processing in direct distribution can effectively reduce environmental impacts by 20% to 48%. Nevertheless, they also indicated that additional improvements, such as better fertilization practices, are still needed to further improve the environmental sustainability of SFSCs. Based on the above discussions, it is hypothesised that:

H1c. SFSCs positively influence environmental sustainability performance.

While the cultural pillar represents a new aspect of regenerated sustainability, it also contributes to the traditional three pillars (Astara 2014). The same feature was also confirmed by Tweed and Sutherland (2007), and they provided a framework under which culture is connected with the three pillars of sustainability. The cultural capital (Bourdieu 1986) could be preserved for future generations (UNIDO 2005). According to Torjusen et al. (2004), consumers responded that SFSCs have increased their knowledge about local agriculture, and it allowed them to give feedback to farmers. Based on the above discussion this study posits that,

H1d. SFSCs positively influence cultural sustainability performance.

Governance is important in delivering the Sustainability Development Goals as declared by many researchers (Biermann et al. 2014; Williams et al. 2018). FAO (2017) confirmed the necessity of creating a governance system to measure, monitor and guide sustainable agriculture. Good governance can help to fight corruption and protect human rights and rule of law. Meanwhile, a major focus of equitable governance is to reduce economic inequality. Khan et al. (2020) suggested the collaboration between the regulatory authority and non-governmental actors for enhancing green practices through “certification schemes” may help to promote a sustainable agenda in supply chain operations. Demartini et al. (2017) highlighted the possible effects of SFSCs on-farm management and food quality and suggested that consumers become the third-party certification bodies and communicate with farmers directly about their needs. Based on these discussions, this study proposes the following hypothesis,

H1e. SFSCs positively influence governance sustainability performance.

### Moral economy

The second main component concerns the moral economy perspective. Initially proposed by a British political economist in 1971, the term “moral economy” was originally defined as “a popular consensus as to what is legitimate and what are illegitimate practices, grounded upon a consistent traditional view of social norms and obligations, of the proper economic functions of several parties within the community” (Thompson 1971). The emergence of this concept was caused by the divergence of economic and moral concerns in the political economy (Götz 2015). Therefore, in order to bring questions of morality back into the economic sphere, the concept of the moral economy was proposed and developed to involve concerns for goodness, fairness and justice, rather than solely the modern economic theory.

While moral economy always refers to economic behaviours or arrangements concerned with survival, redistribution, or risk minimisation in early studies (McCarthy 2006), it has been expanded to include extra factors such as pleasure, friendship, aesthetics, affection, loyalty, justice and reciprocity (Kloppenburg et al. 1996). According to Reuter (2018), the moral economy has become a “culture‐specific moral framework of norms, values, and practices of mutual aid that typically have operated within local societies and their food systems”. It has been applied to the entire food supply processes (production, exchange and consumption) in both first and third world settings (Goodman 2004).

Although the concept of the moral economy has been proposed for over a half-century, research investigating its application in agricultural food networks is still an emergent area, with the majority of studies conducted within the last decade. For example, Leiper and Sather (2017) investigated the motivations of both farmers and consumers in participating FMs and attempted to elucidate the shared values and morals among both parties. They organised data collection at 5 FMs and conducted a questionnaire survey with 377 consumers and semi-structured interviews with 17 producers. It was found that the embedded social relationship between farmers and consumers in FMs and other forms of AFNs is a primary characteristic of a moral economy. Moreover, the localism feature of such food systems also contributes to the moral economy.

Meanwhile, Reuter (2018) conducted a case study in Indonesia to evaluate the loss of moral economies caused by the modernization of regional food systems. Through comparing and assessing the developments of a local food system in the central highlands, he found that with the rapid modernization, the investigated area had experienced a significant decrease in biodiversity, food security, and social solidarity. Although it was found that some aspects of the moral economy still exist in the current system, such as the personal trust between farmers and wholesalers and their focus on reputation, a renewal and redesign of the local food system that features more moral economy is demanded. Through the sharing of risks and benefits, an effective moral economy can provide mutual insurance, and hence help to improve farmer livelihoods and the resilience of the food systems. Therefore, the following hypothesis is proposed,

H2. The positive effect of the moral economy on the local food system positively influence consumer’s motivations to participate in SFSCs.

### Chinese context and effective relationship

This study focuses on China. As a unique business concept in the Chinese context, a relationship with business partners is vital to the success of a business. It generally refers to having a personal relationship and trust with someone, which can involve moral obligations and exchanging favours. The essence of Chinese relationship is to build a network of mutually beneficial relationships which can be used for personal and business purposes. The depth of this type of relationships can be much deeper than ordinary business relationships in the west and can involve a fair proportion of personal relationships.

The history of this unique form of business relationship in China, although not fully documented, can be traced back to the era of the Dynasty (Huang 2009). It emerged as a result of cultural implications of the rule of law, to supplement as an additional insurance on trust among Chinese people in personal and business matters. For those involved in a web of Chinese relationship, favours can be much easier obtained from other participants at lower or no cost but may require reciprocations in the future. Reciprocation is an important component in maintaining this relationship. Together with favour, they form the basis of this business relationship. Participants in a web of this relationship can receive good reputations when they are willing to give favours to others, and bad reputations when they failed to provide reciprocation to those, they acquired favours before. Participants with a consistent bad reputation will be excluded from the web and will have more barriers, even compared with outsiders of the web, if they attempt to work with other participants again. Thus, it can be noted that maintaining a good reputation within the web is important to all participants, which can hence explain the reason Chinese people treating this form of business relationship as an additional insurance on trust.

Both the studies conducted by O’Kane and Wijaya (2015) and Giampietri et al. (2016, 2018) have identified the direct communication feature as a primary motivation for customers and farmers’ participation in SFSCs. Customers can regain their trust in food quality and security and have more sense of involvement in local development, while farmers can feel more empowered and equitable. Therefore, the study proposes the following:

H3. The effective relationship between farmers and consumers has a positive effect on their participation in SFSCs.

### Theoretical framework

**Fig. 1:**
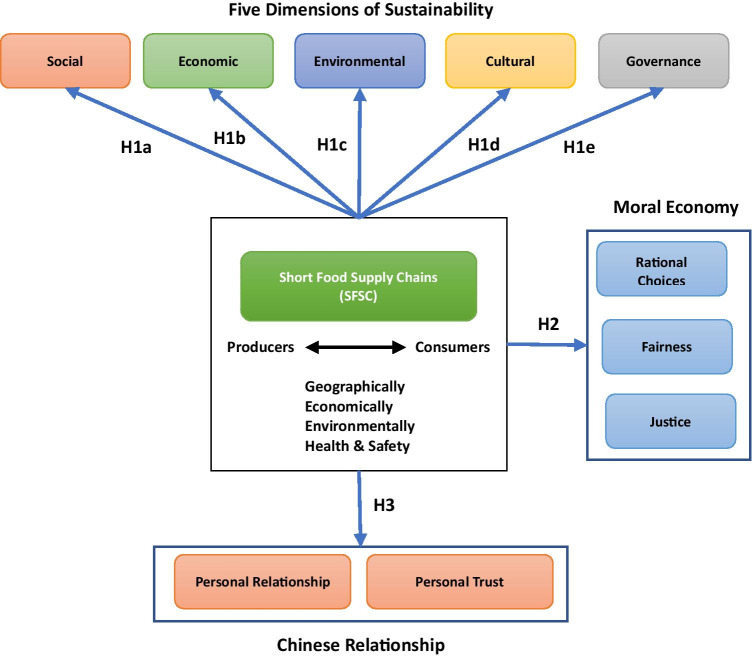
Conceptual Framework

In order to guide and facilitate the empirical investigation into consumers’ attitude towards SFSCs based on the discussions presented in the previous section, a theoretical framework is proposed, as shown in Fig. 1. It can be noted that the designed framework consists of three main components, namely five dimensions of sustainability, moral economy and Chinese relationship. The figure also shows the hypotheses that emerged in the previous section.

## Methodology

This section explains the methodology that was used to empirically test the proposed hypotheses shown in the conceptual framework. The first part introduces the data collection method and presents the survey development. Then, Section 4.2 discusses the target population and sampling techniques, it also justifies the selection of the study location.

### Data collection method

In order to guide and facilitate the collection of data from customers that consume fresh food locally, the survey approach has been adopted as the data collection method in this study. In particular, an online questionnaire was found to be an appropriate method to collect data from the targeted population. One advantage of an online questionnaire is that it can be geographically dispersed (Saunders et al. 2012). Moreover, an online questionnaire allows the anonymity of the respondents, which helps in enhancing the response rate and improving response quality (Zikmund et al. 2013). Given the current context where the whole globe is struggling with the coronavirus, this method also appeared to be the best choice for data collection. The questionnaire was structured into two parts. The first part focuses on the respondent’s demographic information and their buying behaviours towards local fresh food. Part two includes questions related to the research model’s constructs. Specifically, this part sought information on respondent’s attitudes towards SFSCs regarding the sustainability development, moral economy, and buyer-supplier relationship embedded in customers’ buying behaviours. All the survey questions in this part were rating and close-ended questions for the respondent to select the extent to which they agree or disagree.

### Location of study

As outlined in the literature review section, the majority of existing studies focused on SFSCs applications in developed countries. Owing to the localised feature of SFSCs, it would be beneficial to investigate this new form of food supply system in developing country context since it can be easier to implement than conventional industrialised food supply chains. Occupying nearly 22% of the world population, China is a developing country where agriculture attracts paramount attention and concern from the government and the public (Lu et al. 2015). While the current food supply system in China has gained tremendous success, it suffers criticisms from many aspects, most notably, the pollution and safety issues. The large-scale usage of chemical fertilizer and lack of awareness of environmental sustainability has been a prominent issue for many decades (Quan and Liu 2002; Jiao et al. 2017; Wu et al. 2018). Meanwhile, multiple severe food safety crises that happened within the past two decades also impairs the consumers’ trust in the existing food supply system (Macartney 2008; Foster 2011). Owing to these two most prominent issues of the current food supply system in China, it can be noted that a reformation of Chinese agriculture that can improve its sustainability would be beneficial. Thus, China was selected in this study to represent the research context of developing countries.

Ranking the fourth in national land area, China has 23 provinces, 4 municipalities and 5 autonomous regions. While nearly all these regions are agriculturally based, it is unrealistic to conduct a macro-level study covering all these areas. Therefore, in order to seek the balance between the complexity and feasibility of data collection, a pilot city that possesses the essential features of the majority of Chinese areas was selected to conduct this study. Xinxiang city of Henan province was hence selected as the data collection location of this study. This is because Xinxiang has a mixed urban-rural geography, which is more convenient for performing short food supply chains. Moreover, it has been reported that 29 new farmer markets and other forms of SFSCs have been built in this city in the last two years (Rural Planning Bureau of Xinxiang 2017). Therefore, it provides sufficient venues to conduct the data collection.

The survey was constructed using Qualtrics and distributed through WeChat, a very popular social mobile phone application that almost all Chinese people have been using nowadays. With the functionality of this application, the online questionnaire can hence be easily distributed among customers. It should be noted that the distribution was restricted within the pilot city, Xinxiang, to ensure the availability of various forms of food supply systems. The data collection for this study took place between March-April 2020.

## Results and discussion

This section presents the findings from the survey questionnaire. The findings are divided into four subsections, focusing on the descriptive analysis of the demographic information and the evaluation of the three sets of hypotheses.

### Descriptive statistics

**Fig. 2:**
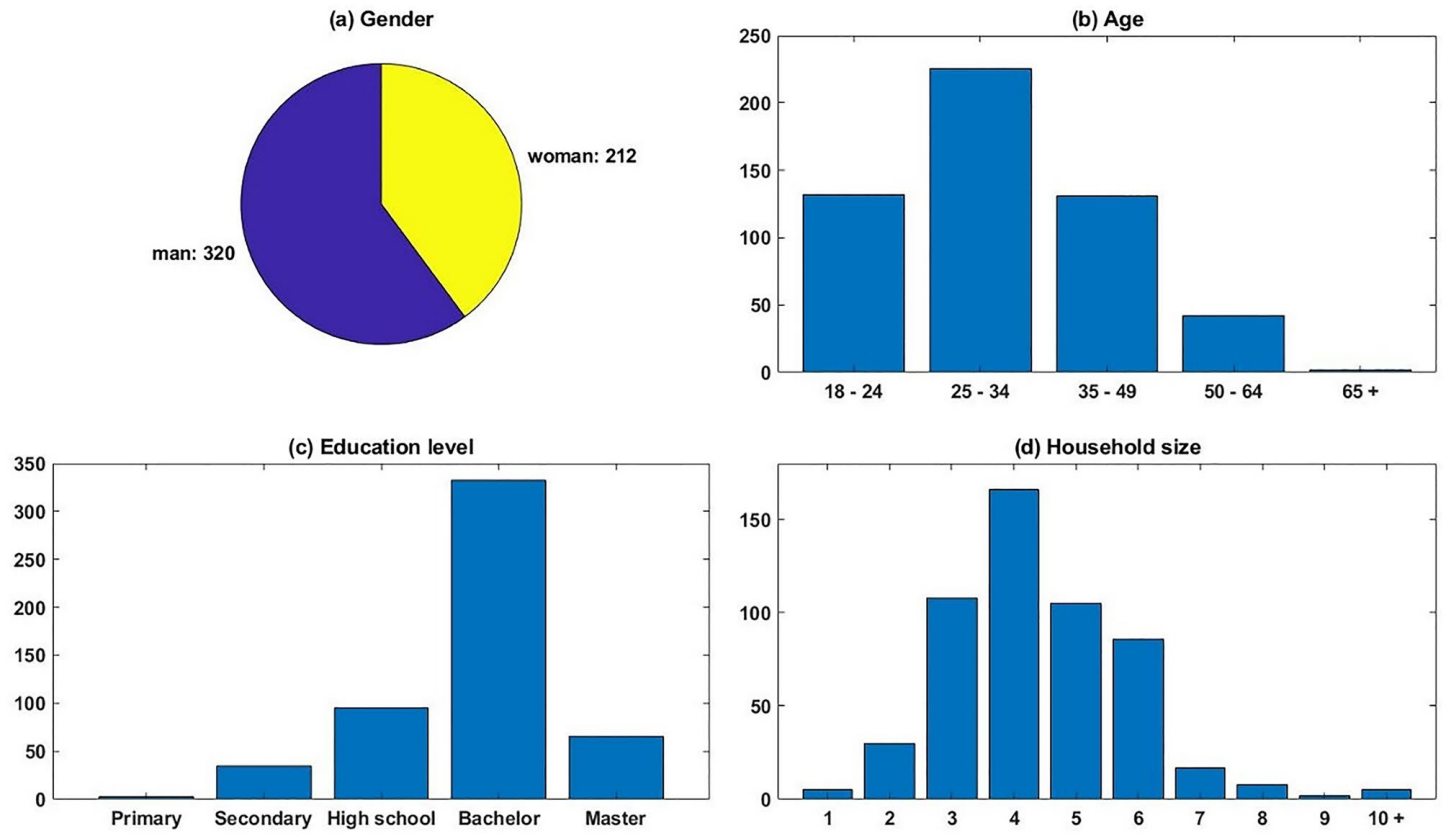
Demographic information

With the World Wide Web becoming fully accessible to people in the 21st century, Sheehan (2001) calculates that the average rate of return of e-mail survey has reached 24 percent. Sellitto et al. (2020) however stated that about a 20 percent response rate is satisfactory for an e-mail survey. In order to test the hypotheses with a sufficient sample, the Qualtrics based survey questionnaire link was distributed through the Wechat app to 10 local Wechat groups where each group had between 200 to 300 members (around 2500 in total) at that time of circulation. This finally resulted in a total of 532 valid responses, representing a response rate of around 21 percent. The basic demographic information of the participants is summarized in Fig. 2.

As illustrated in Fig. 2a, the gender ratio between male and female respondents is approximately 1.51. As the respondents were selected randomly during the distribution of questionnaires, the gender ratio hence cannot indicate a higher proportion of male customers. However, it does validate that many Chinese males were also involved in food shopping activities.

The distribution of respondents’ age is shown in Fig. 2b. It can be noted that customers aged between 25 y/o and 34 y/o have the largest accumulated number of 225, which occupies 42.3% of all participants. The number of participants aged within 18 – 24 y/o and 35 – 49 y/o segments are almost the same, with 132 and 131 participants, respectively. Elder people occupy a relatively smaller proportion, only 42 participants aged between 50 and 64, and only 2 were older than 65. From the age distribution, it can be noted that younger and middle-aged adults (18 – 49 y/o) form the majority group of this survey, with 488 participants and occupies a proportion of 91.7%. This phenomenon can be easily interpretable as this survey was distributed using WeChat, a mobile phone-based application. It should be noted that although older Chinese people tend to also have their own mobile phones, they are more concentrated on the basic functions and are less frequently use these additional applications. However, for the younger generation and middle-aged Chinese people, WeChat has been the main social platform during recent years, which allows the questionnaire to be easily distributed to them. Therefore, although the age distribution cannot be directly linked to the composition of customers in selected types of SFSCs, it helps to justify the respondents who participated in this online survey.

The education levels of the respondents were also acquired from the survey. As illustrated in Fig. 2c, a sum of 398 participants went to universities, which occupies a proportion of 74.8%. Meanwhile, 96 participations graduated from high school, occupying a proportion of 18.0%. 35 participants graduated from secondary school and 3 respondents only went to primary school. From this distribution of education level, it can be noted that the majority of participants have completed the nine-year compulsory education, which was officially regulated by the Chinese government’s policy since 1986. This distribution also correlated with the participants’ age. As most participants were younger or middle-aged, they all benefit from this policy and have acquired a relatively high education level.

From the distribution of household size shown in Fig. 2d, it can be noted that most participants have two or three generations (87.4% for household size between 3 and 6). This also correlates with the age distribution, as it is a very common phenomenon that middle-aged couples have one or two children, and may live with some of their parents, who help them to take care of their children.

**Fig. 3:**
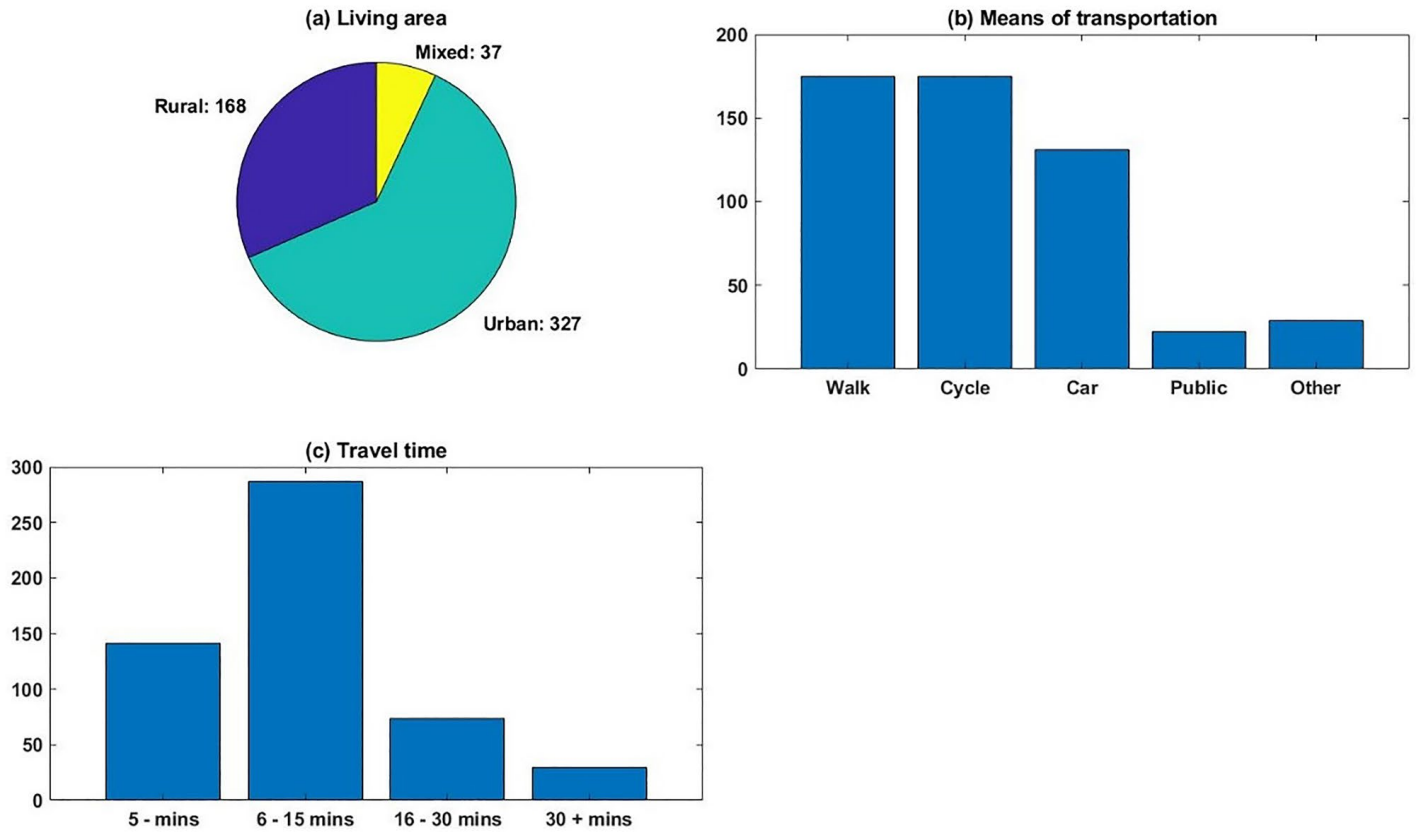
Transportation information

The descriptive results of transportation information are shown in Fig. 3. It can be noted from Fig. 3a that most respondents live in an urban area, with 327 counts and occupying a dominant proportion of 61.5%. Meanwhile, only 168 participants (31.6%) live in a rural area, and the remaining 37 people live in a mixed area. The larger proportion of urban residents can be caused by two main reasons, the first cause is that people living in a rural area have a higher tendency of owning farmland and growing their own agriculture products, which in turn reduce their need to purchase from the food markets. Moreover, this survey was disseminated using a chatting app on mobile phones, and rural residents tend to show less possession and attention to these technology products. Thus, the likelihood of having rural residents participating in this survey may hence be restricted. Nonetheless, the higher proportion of urban participants reflects the actual situation of the Chinese context.

The distribution of respondents’ means of transportation is illustrated in Fig. 3b. It can be noted that walk and cycle are the two most favoured transportation means by these participants, each has an accumulated number of 175. Meanwhile, third-ranking is shopping by car, which has 131 participants and occupying a proportion of 24.6%. Public transportation is the least preferred option with only 22 respondents (4.1%), while the remaining 29 participants categorized their transportation means as others. From the distribution of transportation means, it can be noted that since walk and cycle are preferred by most respondents, they tend to live in convenient distance to their selected shopping venue, which could be a prominent factor for their shopping preference.

Along with the means of transportation, the distribution of travel time is shown in Fig. 3c. It can be noted that the majority of respondents spent between 6 and 15 minutes travelling for food shopping, which occupies a proportion of 53.9% (287) of all participants. Meanwhile, 141 participants (26.5%) travelled less than 5 minutes and 74 respondents (13.9%) travelled between 16 and 30 minutes. Only 30 participants (5.6%) travelled for more than 30 minutes. The high distribution in short travel time ranges correlates with the finding from these respondents’ transport means that they prefer to shop at nearby venues.

**Fig. 4:**
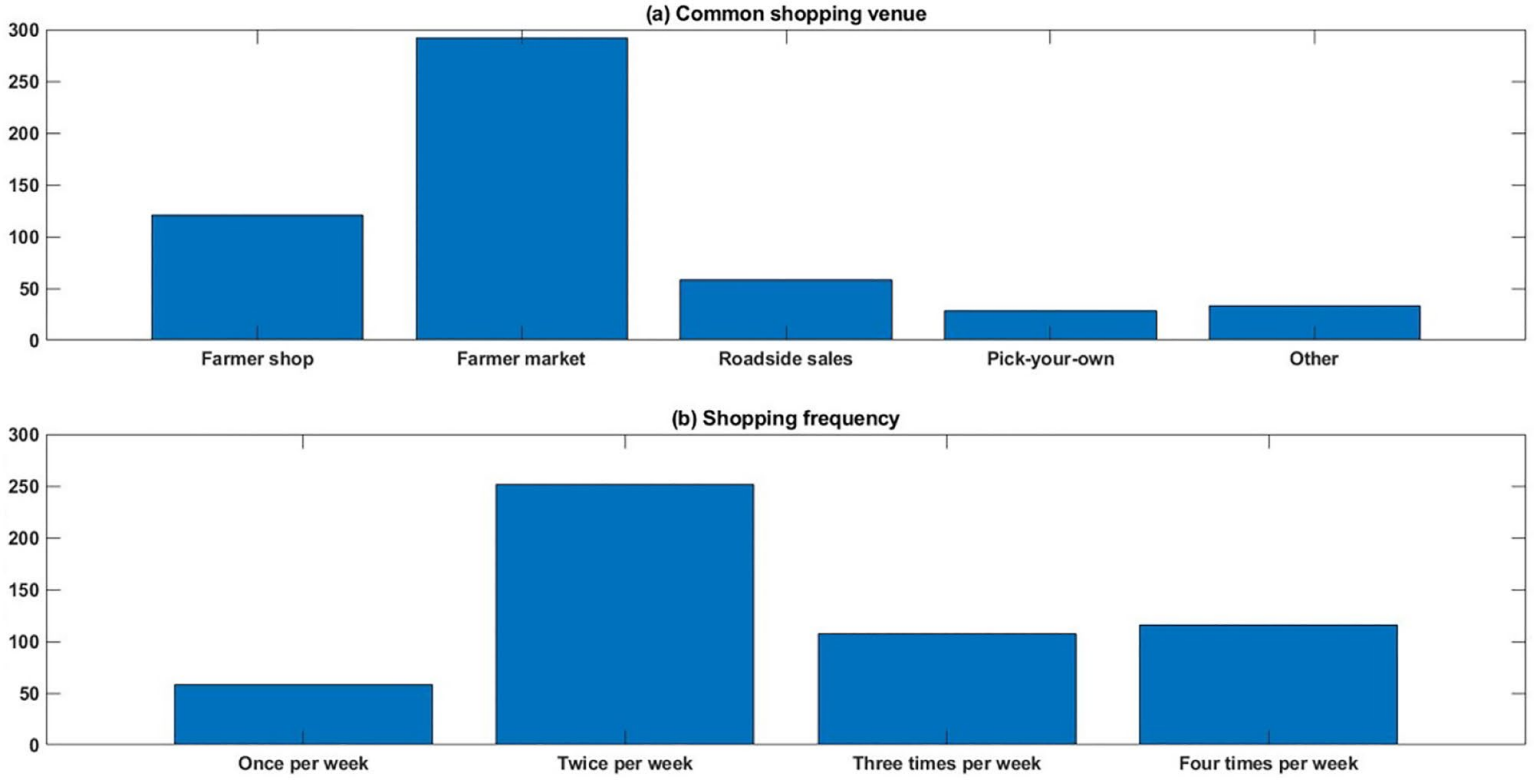
Shopping information

Figure 4 presents the results of respondents’ preference for shopping venue and frequency. The preferred shopping venue is illustrated in Fig. 4a. As shown in Fig. 4a, the farmer market was the most preferred form of shopping venues, with an accumulated number of 292 respondents, occupying a proportion of 54.9%. Meanwhile, the farmer shop is the second favourite shopping venue, with 121 participants (22.7%). The remaining 119 participants preferred to shop in roadside sales (58), pick-your-own (28) and other forms of the venue (33).

Meanwhile, their shopping frequency was also summarized in Fig. 4b. It can be noted that the majority of respondents (251) preferred food shopping twice a week. 223 respondents shopped more frequently, three times (107) or four times (116) a week, and 58 participants shopped only once per week. As the percentage of people shopping more than once a week occupies a dominant proportion of 89.1%, it can be noted that most of these respondents have a relatively high requirement for the freshness of food. Meanwhile, this frequent shopping behaviour also reveals the fact that travelling to these shopping venues is not a constraint for them.

From the presented demographic information of respondents, three general trends can be noted. Firstly, a high correlation was found between their age and education level. It should be noted that younger participants tend to hold higher education level. Among participants older than 50 y/o, the proportion of graduating from high school and below is much higher than younger participants. This phenomenon reveals the fact that education is occupying a very important position in the current Chinese culture, which is in a rapid transition from an agriculture-based developing country to a modern developed country. From the perspective of the majority of Chinese people, receiving higher education has become one of the prominent, if not the most prominent, option to improve their livelihood and overcome poverty. Secondly, regardless of how they travel to the shopping venue, these participants would very seldomly spend over 30 minutes on the road. This indicates that the convenience of shopping is very important to their decision-making. Thus, their locations should be carefully selected when designing new shopping venues. Thirdly, while there are plenty of farmer markets and farmer shops in the pilot city already, pick-your-own is relatively rare and only has very limited instances. From existing studies investigating pick-your-own in developed countries, it can be noted that this type of SFSCs has received extensive research focus and government or local authorities’ support. Many researchers have found that pick-your-own can benefit tourism and possess additional cultural values, both of which are relatively unique features comparing with the other forms of SFSCs (Hara et al. 2013). Therefore, extra attention should be drawn to this type of SFSCs, which can potentially generate more benefits in the longer term.

We then conducted a reliability analysis to test each variable and Cronbach Alpha values of the constructs are shown in Table 1. As it is evident from the table, the Cronbach alpha value for all the variables was >0.70 which is considered to be acceptable.

**Table 1:** Reliability Scores

Variables	Cronbach Alpha Value (>0.7)	No. of Items
SFSC	0.883	5
Economic	0.921	4
Environmental	0.920	4
Social	0.914	4
Governance	0.846	4
Cultural	0.886	4
Moral	0.915	4
Relational	0.939	4

### Hypothesis evaluation – Five dimensions of sustainability

To facilitate the evaluation of the linkage between SFSCs and the five dimensions of sustainability, the collected survey responses are summarised in Table 2.

**Table 2:** Five dimensions of sustainability

Variables	Mean	Dev. St.	1	2	3	4	5
			No.				
Social dimension							
I prefer buying locally as it offers me the opportunity to have social interaction with farmers.	3.78	1.22	32	46	141	103	210
I feel my voice has been heard if I buy locally.	3.61	1.29	45	56	145	99	187
I think buying locally can improve food quality and farmers empowerment simultaneously.	3.82	1.22	34	39	133	111	215
I feel I contribute to improving the farmer’s socio-economic conditions.	3.90	1.19	29	35	126	110	232
Economic dimension							
I prefer buying locally as I believe farmers get a higher share of the profits.	4.02	1.13	24	25	115	118	250
I think buying locally contributes to the local economy.	4.10	1.11	21	25	102	114	270
I prefer buying locally as I can access high-quality food at a fair price.	4.08	1.10	21	25	104	122	260
I prefer buying locally because I think it improves the quality of farmers’ lives.	4.08	1.09	15	31	114	110	262
Environmental dimension							
I feel contributing to improving local biodiversity by buying locally.	3.77	1.25	38	41	137	103	213
I feel in power to influence farmers to use sustainable farming practices.	3.81	1.23	38	34	131	118	211
I believe that locally produced food contains less packaging waste.	4.01	1.14	24	29	109	124	246
I prefer buying locally because the carbon footprint is low.	3.99	1.20	28	38	105	103	258
Cultural dimension							
I believe in buying locally as I have a great understanding of locally grown agricultural products.	3.85	1.20	29	40	134	110	219
I prefer buying locally as I can touch and feel the products and choose the ones I prefer.	4.13	1.04	18	16	101	140	257
I choose to buy locally because I have greater trust in locally grown products.	3.95	1.13	24	27	128	125	228
I prefer buying locally as I get a chance to bargain.	3.80	1.20	32	37	141	117	205
Governance dimension							
I feel more confident in buying local food products if there is a certification body reinforcing product quality.	4.21	1.06	18	15	103	99	297
Buying locally offers an opportunity for customers to participate in food quality requests and check.	4.00	1.13	25	25	115	128	239
I think interactions between farmers and consumers can be an alternative to certification bodies in food supply chains.	3.75	1.28	42	48	120	113	209
I think consumers can help to deal with unsold products by buying them at a discounted price.	3.87	1.15	25	34	139	120	214

Highly disagree, 2: Disagree, 3: Neutral, 4: Agree, 5: Highly agree

As shown in Table 1, each sustainability dimension has four constructs concerning different aspects of customers’ attitudes and motivations towards SFSCs. It can be noted that the economic factors have received the highest approval from survey participants, with an average mean value of 4.07 and an average standard deviation of 1.11. The large mean and small standard deviation values indicate that almost all participants highly agree with the statements about the economic motivations, and they believe in the positive benefits of participating in SFSCs on economic sustainability. Among the four economic constructs, most participants agree with the second factor that “I think buying locally contributes to the local economy”. Meanwhile, the largest dissension occurs in the social dimension. The average mean value of social factors is 3.78, which is the smallest among the five dimensions, indicating that these variables receive less approval from the participants. Meanwhile, these social factors also have the largest average standard deviation (1.23), which reveals that the participants’ opinions on these constructs vary more significantly than other sustainability dimensions. It should be noted that the fourth construct “I feel I contribute to improving the farmer’s socio-economic conditions” is the social factor that receives the highest approval. Regarding the environmental dimension, the most approved construct is the third one, “I believe locally produced food contains less packaging waste”. Meanwhile, the first construct of improving biodiversity is the factor that receives the least approval from the participants. Among the four constructs of cultural dimension, it has the variable that receives the second-highest approval among all variables concerning the five dimensions of sustainability, which is “I prefer buying locally as I can touch and feel the products and choose the ones I prefer”. It can be easily interpretable as comparing and picking from the same product group is a very common behaviour in Chinese culture. Owing to the relatively loose produce control, the qualities of agricultural products can vary significantly. Thus, Chinese consumers prefer to choose the products by themselves. Moreover, it should also be noted that this variable also has the smallest standard deviation among all variables, meaning most participants hold the same opinion towards this factor as well.

The construct that receives the highest approval belongs to the governance dimension, “I feel more confident in buying local food products if there is a certification body reinforcing the product quality”. This construct also has the second smallest standard deviation value among all variables. This reveals the fact that nearly all participants are aware of the demand for a quality monitor and control from official authorities. The lack of this quality control scheme is also one of the most imminent problems faced by SFSCs in China. The consumers’ preference for quality certification is also reflected in the third construct, “I think interactions between farmers and consumers can be an alternative to certification bodies in food supply chains”. This construct receives the least approval from participants and the consumers’ opinions towards this factor varies significantly as well, meaning participated consumers still prefer quality control from official authorities. This finding also coincidences with the findings in a previous study, which investigated the SFSCs in the pilot city from the farmers’ perspective (Wang et al. 2018). According to the interviewed farmers, only agriculture products from greenhouse receive regular checks from the local government, all other produces receive no external monitors and they can sell these products to consumers freely without any regulations. Thus, it can be noted that developing a quality control scheme for products sold in SFSCs can be vital to the success of promoting SFSCs in the Chinese context.

To further verify the conclusions drawn from the descriptive analysis and to test the hypotheses H1a-H1e, a correlation analysis was performed. The findings of the correlation analysis are shown in Table 3. It is quite evident that short food supply chain is positively and significantly correlated with five dimensions of sustainability namely, social (.742**), environmental (.819**), economic (.740**), cultural (.738**) and governance (.665**), all significant at .01 level. Hence, our findings confirm the hypotheses H1a-H1e which advocates that SFSCs positively influence sustainability performance. Our first set of hypotheses thus provides empirical evidence to SFSC and sustainability linkages as suggested by several researchers such as Torjusen et al. (2004), Giampietri et al. (2016, 2018), Balázs et al. (2016), Tasca et al. (2017), Demartini et al. (2017), and Kumar et al. (2019).

**Table 3:** Correlations (Pearson Correlation)

Variables	SFSC	Social	Economic	Environmental	Cultural	Governance	Moral	Relational
SFSC	1							
Social	.742^**^	1						
Economic	.740^**^	.798^**^	1					
Environmental	.819^**^	.810^**^	.817^**^	1				
Cultural	.738^**^	.836^**^	.766^**^	.760^**^	1			
Governance	.665^**^	.772^**^	.707^**^	.703^**^	.802^**^	1		
Moral	.690^**^	.824^**^	.752^**^	.763^**^	.808^**^	.824^**^	1	
Relational	.689^**^	.838^**^	.726^**^	.768^**^	.835^**^	.829^**^	.889^**^	1

*Correlation is significant at the 0.05 level (1-tailed); **Correlation is significant at the 0.01 level (1-tailed)

However, there are several significant differences between our findings and existing studies that should be noted. In most existing studies, the social pillar of sustainability is found to be the most prominent factor that motivates people’s participation in SFSCs. The impacts of some typical social benefits, such as direct interaction (Hinson and Bruchhaus 2008; Sgroi et al. 2014); Tudisca et al. 2015; Demartini et al. 2017) and improved product quality (O’Kane and Wijaya 2015; Engelseth 2016; Leiper and Sather 2017) are widely acknowledged. However, based on the collected responses in our survey, it was found that while most respondents also agree with the social benefits of SFSCs, the influence of this pillar is less prominent and hence is not the main driver motivating their participation.

Another huge difference lies in the economic pillar aspect. The economic benefits of SFSCs is a bit ambiguous among existing studies, which is highly dependant on the form of SFSCs. For instance, studies focused on FMs found a positive influence on economic sustainability (Watts et al. 2011; Jones and Bhatia 2011; Benedek et al. 2017). Meanwhile, studies investigating CSA found the economic benefits of SFSCs are less evident (Janssen 2010; Balázs et al. 2016). As concluded by Charatsari et al. (2020), the potential economic benefits of participating in SFSCs tend not to be the main motivation of their participation. However, findings from this study suggest that the economic pillar is the most prominent motivating factor among the respondents. Interestingly, the importance of economic benefits was also confirmed in another study conducted in China (Zhang et al. 2019).

From the above two major differences, while we still lack sufficient evidence to reach a convincing conclusion, it can be inferred that the underlying cause is related to the context of the studies, e.g. developing and developed countries. Since most existing studies were conducted in developed countries, the income and livelihood of participants are much better than in developing countries. Thus, the participants tend to focus less on the price of the commodities and pay more attention to other added values, such as social interaction and feeling of contribution to the local community. However, in developing countries, the margin of profit is smaller and hence economic benefits dominate the choice of most participants. As the proposed study is conducted in a mixed urban-rural city, where the average income of citizens is not very promising, the reversed impacts of the social and economic pillar to existing studies can hence be easily interpretable.

### Hypothesis evaluation – Moral economy

The survey responses concerning the linkage between SFSCs and moral economy are summarised in Table 4. It can be noted that the fourth variable, “I think buying locally can support farmers and local development”, is the most approved factor concerning the moral economy. The standard deviation of this variable is also among the smallest as well. It should be noted that while the mean values of the other three variables are still larger than 3, they are still relatively smaller than other variables under different hypotheses. The second variable, “I trust in buying locally because I can check the good standards of animal welfare”, has the smallest mean value among all variables. This means that the participants are less approval of this variable and are more doubt about the benefits of SFSCs on animal welfare. It should be noted that the most common types of SFSCs in China are farmer shop and farmer market, both of which are major in selling crops. Thus, the relatively lower level of approval of this variable can be easily interpreted.

**Table 4:** Moral economy

Variables	Mean	Dev. St.	1	2	3	4	5
			No.				
Moral economy							
I prefer buying locally as it offers fair trade for local farmers.	3.95	1.10	22	24	134	131	221
I trust in buying locally because I can check the good standards of animal welfare.	3.74	1.22	40	32	146	120	194
I think buying locally can address environmental concerns.	3.82	1.21	34	34	141	110	213
I think buying locally can support local farmers and local development.	4.03	1.10	21	23	117	128	243

Highly disagree, 2: Disagree, 3: Neutral, 4: Agree, 5: Highly agree

Findings from Table 3 shows that SFSC is positively and significantly (0.01 level) correlated (.690**) with a moral economy. Thus, our finding also supports H2, i.e. moral economy positively influence consumer’s motivations to participate in SFSCs.

### Hypothesis evaluation – Chinese relationship

Finally, Table 5 presents the survey responses about the Chinese relationship in SFSCs. It should be noted that the mean values of all four variables are very close, ranging from 3.83 to 3.88, indicating that the respondents’ opinions towards these variables are similar. The third variable, “I think buying locally helps to increase social inclusivity”, is the least approved factor with the smallest mean value and largest standard deviation. However, the relatively small mean values of all four variables indicate that while most participants tend to agree with these variables, their opinions towards the Chinese relationship in SFSCs are less prominent. Thus, it should be noted that the linkage between the Chinese relationship and SFSCs is less evident. The correlation analysis findings (Table 3) show that relational variable is also positively and significantly correlated (0.689**) thus supporting our third hypothesis H3, i.e. the effective relationship between farmers and consumers has a positive effect on their participation in SFSCs. This was also echoed by a few researchers such as O’Kane and Wijaya (2015) and Giampietri et al. (2016, 2018).

**Table 5:** Chinese relationship

Variables	Mean	Dev. St.	1	2	3	4	5
			No.				
Chinese relationship							
I prefer buying locally because it appears more trustworthy due to direct communication with producers.	3.86	1.17	28	31	145	112	216
I prefer buying locally as I think it’s important to develop a personal relationship with producers.	3.88	1.18	29	36	126	121	220
I think buying locally helps to increase social inclusivity.	3.83	1.21	33	39	127	118	215
I think the personal relationship motivates farmers to produce healthy and safe food.	3.87	1.19	32	27	139	113	221

Highly disagree, 2: Disagree, 3: Neutral, 4: Agree, 5: Highly agree

## Conclusion

This study aims to investigate the customers’ attitudes towards participating in SFSCs. To ensure the validity and generalization of the findings, a pilot study was implemented in a city that possesses mixed urban-rural geography and belongs to the largest agricultural-export province in China. A novel theoretical framework consisting of three fundamental theories was first proposed to guide this study. Afterwards, a questionnaire was designed following the proposed theoretical framework that resulted in 532 completed responses.

Through the analysis of the demographic information, it was found that the farmer market is the most preferred form of SFSCs by the participants. Meanwhile, farmer shop, roadside sales and pick-your-own are also favoured by some participants. Moreover, while the means of transportation to the shopping venues vary among these participants, they tend to prefer spending less than 15 mins on travel, indicating that convenience is a prominent factor in their motivations. From the perspective of hypothesis evaluation, all hypotheses H1a-H1e, H2 and H3 were found to be supported by the empirical data. It can be however noted that among the five dimensions of sustainability, the economic pillar is the most approved factor by the participants as evident from the descriptive analysis of the data. This can be easily interpretable as compared with other factors, the economic benefits are more intuitive. An interesting finding is that the social pillar has received the smallest average score, indicating the participants are less approval of those social variables. This finding is different from studies conducted in developed countries, where they found that the social connection, especially direct communication with farmers, is a major motivation for their participation in SFSCs. Nonetheless, it should be noted that the average score of all four social variables are still larger than 3, indicating that while the influence of the social pillar may be less prominent than others, most participants still hold positive attitudes towards it. With an average mean value of 3.92 across the 20 variables from the five dimensions of sustainability, it can hence be noted that the first set of hypotheses is validated. These were further verified through the correlation analyses which showed a strong and positive correlation. Meanwhile, although the average mean values of variables concerning the moral economy and Chinese relationship are smaller (3.88 and 3.86), these values indicate that the proposed variables are still agreed by most participants. Thus, it can be noted that the moral economy and Chinese relationship have positive effects on the consumers’ participation in SFSCs, which validates the second and third hypothesis.

This study provides several theoretical contributions. First, this study is one of the first studies that attempts to empirically validate the relationship between SFSCs and five dimensions of sustainability, which has been largely missing in the existing literature. Second, this is also perhaps the first study that attempts to combine the moral economy and relationship aspects and provides empirical validation. Third, the framework proposed in this study is novel and provides a broader understanding of consumer attitudes towards SFSCs and Sustainability. Finally, this study also adds to the limited literature on SFSCs in a developing country context.

From practical contributions perspective, the findings of our study are quite useful for policymakers as it provides an understanding of the consumer attitudes towards SFSCs, particularly when viewed from sustainability lense. This will help them in designing local and regional policies to promote more sustainable farming practices and encourage farmers to adopt SFSCs practices to serve local needs. Findings would also benefit farmers who are often less resourced to understand the shifting consumer trend and are hesitant to participate in SFSC activities. Consumers are slowly preferring more healthy and safe food amid food scandals, health problems and increasing awareness towards sustainability practices. The current COVID-19 pandemic has further shown the benefit of adoption of SFSCs as global supply was disrupted due to lockdowns and also the demand for healthy, safe and fresh fruits and vegetables has grown significantly.

There are also some limitations to the current study that should be addressed. Firstly, as the selected pilot city is within the largest agricultural province in China, the coverage of farming can be larger and more distributed than in other provinces. Thus, it can potentially be easier for consumers in the pilot city to get access to SFSCs. Secondly, although WeChat helps to distribute the questionnaires more conveniently, it may also restrict the involvement of elder people. This phenomenon is also reflected in the age distribution of the participants, which is more centralized to the range of younger and middle ages. Thirdly, although some preliminary comparisons have been implemented between the findings from this study and existing studies in developed countries, it should be noted that more thorough comparisons are needed, especially based on data collected from the same questionnaire.

Following the limitations, future research can be implemented in three directions. The first direction is to replicate the study in other pilot cities, carefully chosen from other Chinese provinces with a different agricultural composition to improve the generalisation of the findings. Moreover, future studies can also focus on conducting interviews with consumers to get a greater understanding of their attitudes towards SFSCs in addition to the survey questionnaires, thus adopting a mixed-methods approach. The five dimensions of sustainability have not been extensively explored in the existing literature and hence this opens up avenues to further explore this in future studies in other contexts as well. Finally, this study can be expanded to other developing countries that heavily rely on the agricultural sector and where SFSCs are on the rise such as Brazil, Vietnam and Thailand. Moreover, a comparative study between developed and developing countries can further investigate the difference in SFSCs adoption.

## Data Availability

On request.
